# Understanding the effect of organizational innovation environment and customer participation in virtual communities on customer creativity: A study of mediating and moderating influences

**DOI:** 10.3389/fpsyg.2022.913525

**Published:** 2022-10-27

**Authors:** Mina Ge, Jamal Khan, Yuan Li

**Affiliations:** ^1^School of Business Administration, Zhejiang Shuren University, Hangzhou, China; ^2^Institute of International Studies, Shandong University, Wenhua Xilu, Weihai, China; ^3^School of Northeast Asia Studies, Shandong University, Wenhua Xilu, Weihai, China

**Keywords:** innovation environment, creative self-efficacy, positive emotions, customer creativity, online community

## Abstract

The importance of enterprises significantly improving their innovation performance by working closely with customers throughout the innovation process has been emphasized in recent literature. However, the role of organizational innovation environment in customer creativity and the impact of customer knowledge matching on the innovation environment are not sufficiently explored. Based on the Social cognitive theory and Flow theory, his investigate the factors that influence enterprise innovation performance in the context of customer participation in innovation, as well as how businesses can foster an online innovation environment that fosters customer creativity. The mixed-methods study, which combines qualitative and quantitative research, is used to create the scale item that measures the study’s main variables. Structural equation modeling and bootstrapping estimation was performed on survey data collected *via* questionnaire from 392 consumers who participated in online service innovation in an online virtual brand community between June 2018 and May 2019. The study obtain the following main results. First, the innovative environment of customer online participation in service innovation fosters customer creativity. Second, two mediation interactions exist: creative self-efficacy and positive emotions, both of which partially mediate the relationship between customer creativity and the innovation environment. Third, while customer knowledge matching influences the intermediary effect of creative self-efficacy on innovation environment and customer creativity, it has no effect on the intermediary effect of positive emotion on innovation environment and customer creativity.

## Introduction

The external environment of business is subject to rapid change in the current age of knowledge and information, and businesses must remain creative and innovative which are complementary processes, in order to gain and maintain competitive advantage (Foss et al., 2011; Hughes et al., 2018; Huang and Khan, 2022). Economists have traditionally viewed product manufacturers as the beginning of the innovation process. However, researchers who examine technological and organizational change are skeptical of this viewpoint, believing that assuming the manufacturer as the sole source of innovation would severely limit the overall picture of the innovation process (Zhuang et al., 2019). Thus, research emphasizes the importance of businesses’ existing knowledge and technological bases in facilitating innovation (Ardito et al., 2020), and suggests that businesses must constantly acquire knowledge from outside sources (also known as openness) to meet the knowledge needs of their innovations (Zhuang et al., 2019; Ardito et al., 2020). For example, both the “Open-Innovation” model and strategy literature assert that businesses must build networks and relationships with external agents, who are a valuable resource in the current competitive environment, particularly when it comes to the development of new products and processes (Chesbrough, 2006), and use external ideas to supplement their own resources and capabilities (Laursen and Salter, 2006).

Recently, both the user innovation and open innovation literatures have focused on cooperation with customers and has emphasized open service innovation logic, which consider customers as external resource suppliers during the innovation process (Wang et al., 2020). Empirical research pointed out that cooperation has a variety of effects on the inputs to the innovation process as well as R&D expenditures, three of which merit special consideration. The first stems from customer information, which is critical for the advancement of the innovation process (Geilinger et al., 2020). This information could be about new technologies (Amara and Landry, 2005), scientific information and complementary technology to what the R&D team is already managing (Sánchez-González and Herrera, 2014), and information designed to generate new ideas (Amara and Landry, 2005; Zhou, et al., 2022). The second group is concerned with innovation creation and emphasizes that customer participation facilitates the creation of more appealing products and services while requiring businesses to invest less time and money to achieve a specific innovation (Wang et al., 2020). More importantly, such products have a higher chance of commercial success with fewer costs associated with the trial-and-error process, as they allow for the identification of unmet needs that many customers are unaware of (Schaarschmidt et al., 2018). Finally, the third group asserts that customer cooperation reduced innovation investment and increased the efficiency of the innovation process (Zhou et al., 2022).

Furthermore, the marketing literature has emphasized the importance of innovation in understanding the relationship between market orientation (i.e., focusing on both customers and competitors) and firm-level performance. However, it placed a greater emphasis on internal organization, implying that a firm’s ability to connect market orientation to new product development and financial performance may be dependent on its internal structure. While, on the one hand, the user innovation and open innovation literatures place a greater emphasis on the role of the customer in the innovation process, while ignoring the role of the firm’s internal organization. On the other hand, marketing literature provides an incomplete picture of how customer knowledge can be leveraged for innovation purposes.

In addition, customer creativity has received considerable attention from management and marketing scientists, confirming that consumer creativity significantly contributes to product value creation (Rosa et al., 2008), but has remained largely unexplored in the context of organizational innovation atmosphere^1^. That is, what effect does organizational innovation environment have on customer creativity, and what effect does customer knowledge matching have on the innovation environment? In addition to this, little is known about why customers participate in activities on behalf of an organization and how the organization’s innovative environment influences customer creativity (Setyawati et al., 2019). Both research and practice are insufficiently aware of the benefits that customers derive from their engagement activities, as well as how the organizational innovative environment influences their creativity. Thus, for a firm to effectively manage its stakeholder relationships, marketing scholars and managers must first understand customers’ motivations for engaging with a particular firm or brand, which improves customer creativity, and then gain a deeper understanding of the specific objectives that these various customer engagement activities seek to achieve.

Given the critical role of creativity in a firm’s success and the low level of customer appreciation for the value of creativity, it is imperative to measure the factors that impacts creative process antecedents of customer creativity, and how organizational innovative environment influences customer creativity. To this end, this study aims to fill a research gap by identifying the needs of participating customers, to investigate and analyze the driving factors or generation mechanism that can truly improve the innovation performance of enterprises in the process of customer participation in innovation, that is, the formation mechanism of customer creativity. It will look at how businesses can foster an online innovation environment that caters to customers’ three basic psychological needs: autonomy (virtual empowerment), competence (task orientation), and relationship (knowledge sharing), thereby incorporating customer creativity into core competitiveness. It will help marketers gain a better understanding of the factors that contribute to customers’ creativity and engagement in co-creation activities (Deci and Ryan, 2008).

We use social cognitive theory as a unifying theoretical framework to develop a conceptual model that examines a number of factors that affect human functioning and, consequently, the innovation environment of knowledge sharing and customer creativity (Bandura et al., 1999). It integrates personal influences prominently with two substantial developments: self-efficacy and reciprocal interactions. Self-efficacy theorizes and proves the significance of self-efficacy in human behavior, as well as demonstrating that it has a significant personal impact on motivational outcomes. While the reciprocal interactions model (Bandura et al., 1999) defines how learning occurs in a social context as a result of a dynamic and reciprocal interaction between three variables: the individual, the environment, and behavior. According to social cognitive theory, the ongoing interaction of these three sets of variables influences and is influenced by the others, resulting in the formation of human behavior (Young et al., 2005). Additionally, we employ flow theory to clarify customer participation and its intended benefits, as well as elucidates how and why people feel when they are having the most fun. Considering the significance of providing the best possible user experience, the flow notion of flow theory has evolved into a critical component of the theory of optimal experience. Empirical evidence suggests that flow is important to the creative process and is strongly linked to a positive experience. We derive a set of hypotheses from the theoretical discussion. The empirical analysis is based on survey data from 392 consumers who innovated online services and participated in online virtual brand community innovation between June 2018 and May 2019.

This study has the following main contributions. First, this study contributes to the literatures on user innovation and open innovation by highlighting the role of organizational innovation in customer creativity. To our knowledge, this is the first time a theoretical framework based on social cognitive theory that has been used to develop a conceptual model that examines a variety of factors affecting human functioning and, consequently, the innovation climate of knowledge sharing and customer creativity^2^. Second, this adds to the marketing literature by posing the question of how customer knowledge can be used for innovation. As far as we know, this is the first paper to use flow theory to build a theoretical foundation for understanding customer engagement and its intended benefits. This study contributes to areas where empirical research is needed, most notably in explaining the mediating and moderating variables associated with the impact of the innovation climate on customer creativity outcomes. This research has ramifications for understanding of customer enterprise value co-creation and modern enterprise service innovation in the post-epidemic era.

The remaining sections of the article are as follows: section 2 covers the Theoretical Model and Hypothesis. The research design and data used are discussed in Section 3, and the results are reported in Section 4. Finally, section 5 discusses the recommendations for managers.

## Theoretical model and hypothesis

### Social cognitive theory as the theoretical basis for analyzing customer engagement and its targeted benefits

We use social cognitive theory—which advocates for a critical perspective on human behavior—as a unifying theoretical framework for developing a conceptual model that examines a set of factors that influence human functioning and, in turn, the innovation climate of knowledge sharing and customer creativity. Bandura’s social cognitive theory integrates personal influences prominently with two substantial developments: *self-efficacy and reciprocal interactions*.

#### Self-efficacy

The first postulates and substantiates the importance of self-efficacy in human behavior (Bandura, 1977) and demonstrates that it has a significant personal effect on motivational outcomes. Studies have found that individual characteristics have been found to influence self-efficacy. Individuals who are confident in their ability to learn are more likely to engage in cognitive and behavioral activities that promote learning, such as goal setting, implementing effective learning strategies, and monitoring and evaluating their progress (Schunk and DiBenedetto, 2016). In turn, the outcomes of actions, such as perceived goal progress and achievement, as well as environmental inputs (such as witnessing a successful performance or making social comparisons with peers), can influence self-efficacy and motivation.

#### Reciprocal interactions

Reciprocal interactions model (Bandura et al., 1999) established a conceptual framework for how learning occurs in a social context as a result of a dynamic and reciprocal interaction between three factors: the individual, the environment, and behavior. The continuous interaction of these three sets of factors affects and is influenced by the others, resulting in the formation of human behavior (Young et al., 2005). Personal factors include self-efficacy, expectations, self-regulation, and reinforcement (Ozmete and Hira, 2011). Environmental factors are external to an individual and can either support (for example, familial support) or discourage (for example, familial criticism) a particular behavior. They may be social in nature (friends, family, or coworkers) or physical in nature (workplace facilities or living place environment). According to social cognitive theory, individuals anticipate specific outcomes as a result of their behavior and actions. Their behavior has an effect on their actions and environment, while actions have an effect on their thoughts and environment, and environments have an effect on individual’s behavior and actions as well.

### Innovation environment and customer creativity

Knowledge sharing refers to the dissemination of diverse resources among individuals engaged in particular activities. It has garnered a considerable attention as a means to effectively expand the breadth and quality of knowledge, and is widely acknowledge as a key source of competitiveness among businesses (Majuri, 2022). Furthermore, businesses that invest continuously in technological innovation benefit from having access to new technological opportunities by exploring and applying new knowledge to improve their technological capacity, which results in the creation of new processes and products (Ardito et al., 2020; Xin et al., 2021). Numerous empirical studies have established the beneficial effects of an organizational innovation culture and knowledge on employee creativity (Lee, 2018). For example, Amabile et al. (1996) demonstrates that the organizational innovation environment has a significant impact on employee creativity, and coworkers’ support in teamwork accelerates the creativity of individual team members. Likewise, Zhou and George (2001) demonstrated that peer support and knowledge sharing have a significant positive effect on employee creativity. Lee (2018) found that the quality of knowledge sharing is the most important factor in facilitating individual creativity. Men et al. (2019) looked into when and how knowledge sharing helped teams be more creative, emphasizing the importance of cognitive team diversity.

Apart from internal sources, businesses continuously tap and exploit external sources, such as capitalizing on their customers’ knowledge, which is critical in the early stages of the innovation process (Lichtenthaler, 2008). Interaction with customers has long been viewed as a critical precursor to innovation (Von Hippel, 1976). Customers contribute to the process of innovation for two reasons: First, customers benefit significantly from innovation; second, customers possess sticky knowledge that is costly to transfer. Thus, in order to mobilize customer knowledge and incorporate it into the innovative process, organizations will need to collaborate directly with their customers, as this will provide access to knowledge that the producer firm would be unable to produce in-house—knowledge that may be critical to the success of the innovation (Laursen and Salter, 2006). With the rapid advancement of Internet technology, it is becoming increasingly important for businesses to interact with customers in real time *via* platforms like virtual communities^3^. Customer participation in a virtual community improves the performance of service innovation and assists businesses in obtaining relevant information such as customer needs and lowering knowledge sharing barriers (Wang, 2022). Customers primarily contribute to the service innovation process of businesses through knowledge transfer, thereby assisting in the enhancement of service innovation performance (Xin et al., 2021).

Empirical evidence suggests if customers participating in service innovation are given sufficient authorization and trust, they are more likely to advance their own ideas without violating the virtual community’s rules, and to engage in service innovation tasks freely (Vargo and Lusch, 2004). Lusch and Vargo (2006) emphasized the importance of providing timely incentive and affirmation to leading customers. These material incentives can enhance a consumer’s desire for rewards, which in turn increases customers’ participation in service innovation activities. Overall, the enterprise’s innovation atmosphere can help individuals better understand and participate in creative activities. The innovation atmosphere fostered by online enterprises is characterized by three distinct dimensions: knowledge sharing, virtual empowerment, and task orientation. Therefore, we propose that:

*H1a:* Knowledge sharing in an enterprise's online community has a significant positive impact on customer creativity.

*H1b:* Virtual empowerment in an enterprise’s online community has a significant positive impact on customer creativity.

*H1c:* Task orientation in an enterprise’s online community has a significant positive impact on customer creativity in an enterprise's online community.

### Innovation environment, creative self-efficacy, and customer creativity

Creative self-efficacy refers to an individual’s belief in his or her ability to generate creative outcomes (Luthans et al., 2007) or perform specific tasks during the innovation process (Tierney and Farmer, 2002). Using a social cognitive perspective, we hypothesize that an individual’s environment (for example, job characteristics, positive interactions with peers and coworkers) may enhance his or her creativity by increasing creative self-efficacy. Individuals with a high level of creative self-efficacy are believed to be more confident in their abilities to mobilize cognitive resources and more motivated to complete certain task and develop creative ideas (Tierney and Farmer, 2002; Wang et al., 2014). What is more, creative self-efficacy can drive people to overcome barriers and encourage them to seek creative alternatives to successfully complete their responsibilities. In order to maintain creativity, an individual must also make efforts to try new methods and procedures which necessitates creativity involvement and goal orientation, both of which are associated with creative self-efficacy (Tierney and Farmer, 2002).

In support of this view, a substantial amount of empirical research has been conducted to investigate the relationship between self-efficacy and employee creativity. Some studies found that employee creativity is positively associated with creative self-efficacy (Binnewies et al., 2009; Liao et al., 2010). For example, Carmeli and Schaubroeck (2007) concluded that higher perceived self-efficacy influences individuals’ creative involvement at work. While according to Wang et al. (2014), the relationship between transformational leadership and employee creativity is mediated by creative self-efficacy. Other studies found that employees with a high level of creative self-efficacy are motivated by goal mastery (Beghetto, 2006) and creative work (Carmeli and Schaubroeck, 2007). Several studies investigated the relationship between team atmosphere and team performance (Tsai et al., 2015), organizational innovation atmosphere, individual learning ability, organizational commitment, and individual innovation behavior. Among others, Wang et al. (2014) and Meng and Zhichao (2015) investigated the effect of authentic leadership on employee creativity in Chinese firms. In addition, numerous studies have demonstrated that creative self-efficacy has both direct and indirect positive impacts on individual innovation behavior (Beghetto, 2006; Ding et al. 2021). What is more, Ding et al. (2021) concluded that psychological atmosphere advantage has a beneficial effect on creative self-efficacy and willingness to innovate.

Integrating these findings, this study aims to contribute to the growing body of knowledge about creativity by introducing social cognitive theory and empirically investigating the mediating role of creative self-efficacy on customer creativity. Therefore, we propose the following hypotheses:

*H2a:* creative self-efficacy mediates the effect of online innovation climate of knowledge sharing on customer creativity.

*H2b:* creative self-efficacy mediates the effect online innovation climate of virtual empowerment on customer creativity.

*H2c:* creative self-efficacy plays a mediating role between task oriented online innovation climate and customer creativity.

### Flow exchange theory as the theoretical basis for analyzing customer participation and its targeted benefits (innovation atmosphere, positive emotional experience, and customer creativity)

We expect that positive emotions such as pleasure and happiness contribute to individual satisfaction, and that a relaxed and pleasant organizational environment can boost individual attraction and customer loyalty. In this context, flow theory provides an important theoretical foundation for explaining customer participation and its intended benefits. Csikszentmihalyi (1975) developed the flow theory with the primary goal of elucidating the understanding of how people feel when they are having the most fun, and why. Because of the provision of the best user experience, the flow^4^ concept of flow theory became a key component for the theory of optimal experience. Research pointed out that flow plays a critical role in the creative process and is highly correlated with an optimal experience. It allows individuals to transcend their cognitive limitations, allowing them to express their imagination and experiment with new frameworks. In addition to this, flow can also occur during group activities, and group flow is highly dependent on interaction of group members. Collaboration among team members, as well as sharing emotions and feelings with group members, are regarded as critical components of group flow, as they promote synchrony and help the group become more creative.

Among others, emotion is one of the precursors to creativity (Isen and Baron, 1991). A considerable amount of research work has been conducted to inquire the impact of emotions on team creativity, self-efficacy (Chen et al., 2012), consumer decision-making behavior and innovation behavior. While others, for example, Alsughayir (2021) studied the mediating role of emotional intelligence on both job satisfaction and organizational commitment. Previous research pointed out that customers participate in service innovation not only because they have unique product needs, but also because they have unique emotional experience needs (Kessler et al., 2007). These include feelings of achievement, satisfaction, and enjoyment engendered by interaction and cooperation (Prahalad and Ramaswamy, 2004). Consumers’ needs for products no longer remain at the functional level as a result of profound changes in consumption levels and consumption structure. Products and services are increasingly geared toward emotional and spiritual well-being, happiness, and satisfaction. Positive emotional experiences can help individuals develop positive behaviors (Ferreira, 2015). According to the marketing data, purchases elicit emptions in consumers, which influence their sharing and innovation behavior (Kessler et al., 2007). Chen et al. (2012) pointed out that improving customers’ personal experience and knowledge through online interaction can boost their self-efficacy and positive emotional experience. Therefore, we propose the following hypotheses:

*H3a:* positive emotions mediate the effect of innovation climate of knowledge sharing on customer creativity.

*H3b:* positive emotions mediate the effect of online innovation climate of virtual empowerment on customer creativity.

*H3c:* positive emotions mediate the effect of task orientation in an enterprise online innovation climate on customer creativity.

### The moderating effect of customer knowledge matching

As mentioned above knowledge sharing is a fundamental concept of knowledge management that can maximize an organization’s ability to meet the needs of its customers while also generating solutions and efficiencies that provide a competitive advantage (Olowodunoye, 2015). What is more, the impact of knowledge management on individual and business performance is well documented (Dyer and Hatch, 2006). In addition to internal knowledge, it has been identified that external knowledge sources play crucial roles in the innovation process of the businesses (Flor et al., 2018). Utilizing external knowledge and technology enables businesses to draw on the valuable ideas of outsiders to expand their pool of available technological opportunities, thereby enhancing their innovation outcomes (Shi et al., 2020). Previous empirical studies emphasized the importance of external knowledge to incremental innovation capability of businesses (Sun et al., 2020). According to Watson et al. (2018), relationships with external stakeholders, specifically customers, provide firms with access to resources beyond their boundaries, thereby augmenting the organizational knowledge base.

Customer knowledge is a critical consumer construct because it influences the collection and processing of consumer product information and, ultimately, the purchase and the use of products by customers. Researchers have classified customer knowledge into different categories^5^. From a logical standpoint, customers who participate in the enterprise do so primarily through knowledge transfer, assisting the enterprise in improving service innovation performance. It demonstrates that customer participation in the process of enterprises’ service innovation will improve the transfer of customer knowledge, which is beneficial to customer-enterprise interaction. Service innovation activities are carried out by businesses by acquiring and applying customer knowledge, thereby improving their service innovation performance (Wang, 2022). Xu et al. (2014) investigated the effect of customer innovation and customer product knowledge on customers’ individual innovation behavior. Liu et al. (2018) studied the moderating effect of professional success on community online interaction and customer creative quality. Several studies found that customer participation and knowledge management both have a positive impact on innovation performance (Xin et al., 2021; Wang, 2022), with knowledge management acting as a mediator between customer participation and service innovation performance (Xin et al., 2021). Therefore, we propose the following hypotheses:

*H4a:* Customers' knowledge matching moderates the mediating effect of creative self-efficacy on the knowledge sharing in an innovation atmosphere, on customer creativity.

*H4b:* Customers' knowledge matching moderates the mediating effect of creative self-efficacy on virtual empowerment in an enterprise’s online community on customer creativity.

*H4c:* Customers' knowledge matching moderates the mediating effect of creative self-efficacy on task orientation in an enterprise’s online community on customer creativity.

**Figure 1:**
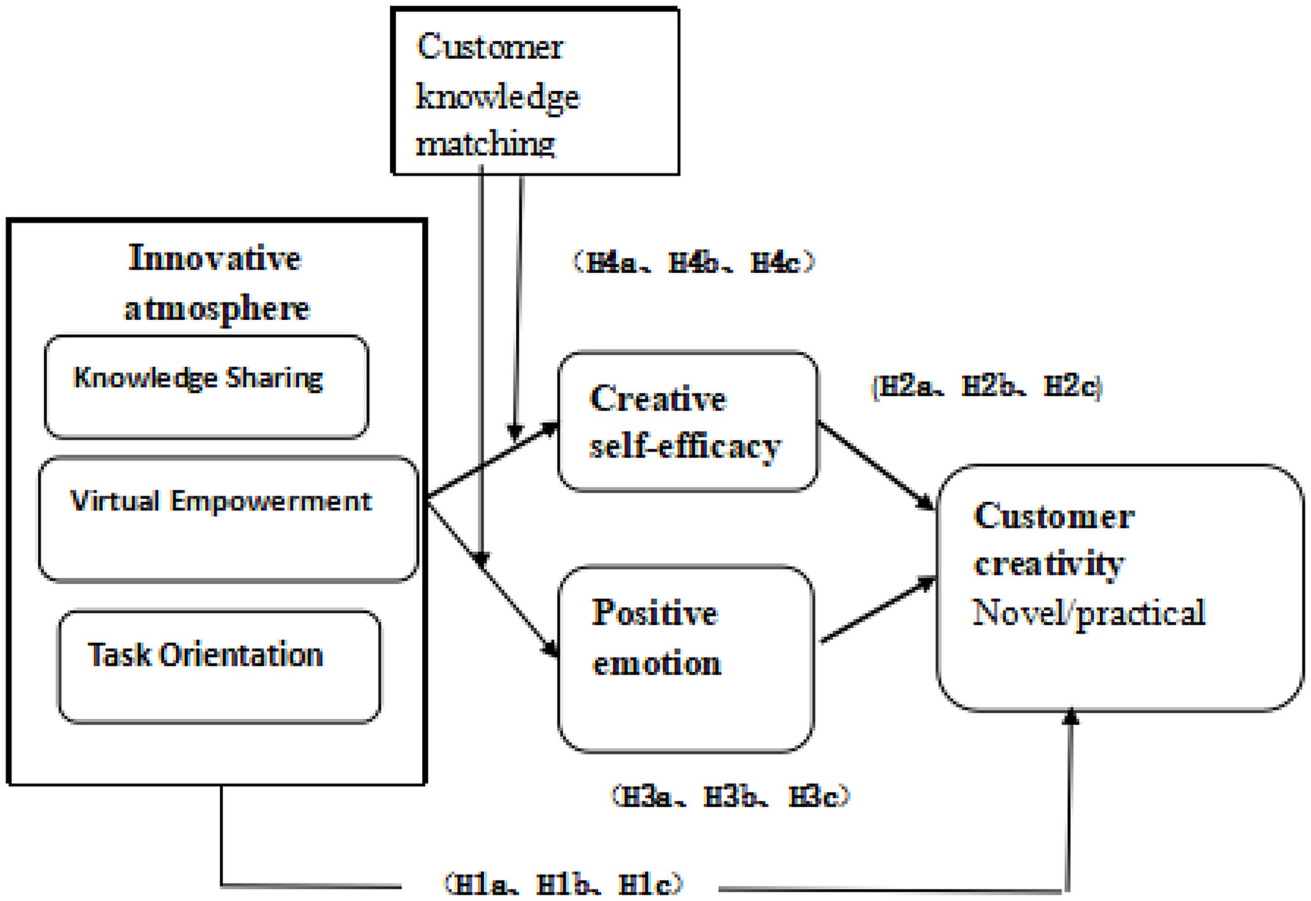
Theoretical model.

This study creates the conceptual model shown in Figure 1 based on the reasoning outlined above.

## Research design

### Sample and procedures

In this study, a structured questionnaire survey with two parts was conducted^6^: basic personal circumstances and scale measurement. Due to the fact that the subject of this study is a virtual community, the first investigation item requires respondents to be consumers who participated in innovation in the online virtual brand community. What is more, each item is measured using a five-level Likert scale in the measurement part of the scale, where 1 represents “strongly disagree” and 5 represents “strongly agree.” After conducting a comprehensive literature review and exploratory case investigation, the initial draft of the questionnaire was created. Following consultation with experts in a variety of research fields, including psychology, organizational behavior, and consumer behavior, we implemented constructive suggestions, made appropriate adjustments, and added and deleted some items, resulting in the second draft of the questionnaire. Pre-testing and purification were used to further improve and modify the questionnaire. The questionnaire, for example, lacked academic terms and annotated key words and concepts. Finally, the formal questionnaire’s final draft was created.

To complete the formal questionnaire, we chose consumers who participated in online service innovation in the online virtual brand community between June 2018 and May 2019, and preliminary screening tests were conducted on questions such as “your understanding of online marketing and degree of participation” and “whether you have participated in the company’s brand virtual community service innovation activities.” We used virtual brand community participatory observation to investigate service innovation and consumer behavior. To complete the questionnaire data collection, we distribute questionnaire on a large scale on the following four virtual brand communities in China: LEGO Online Community, Panshi Network Alliance, MAFENWO self-help travel sharing community, and Haier co-innovation platform.

To complete the questionnaire data collection, this paper uses the questionnaire platform to create formal questionnaires and distribute them on a large scale on the aforementioned virtual communities. After that, a total of 500 formal questionnaires were collected, error messages were removed and 18 incomplete questionnaires were filled out, A total of 392 questionnaires were valid, with an effective rate of 78.4%. The sample distribution in this study is as follows: young people under the age of 30 account for 94.14% of respondents, those with a monthly income of less than 3,000-yuan account for 96.43%, and those with a bachelor’s degree or higher account for 92.84%, which is consistent with the attribute of online service enterprises. Among all participants, 39.09% engage in enterprise-organized online service innovation activities on a regular basis, while 8.14% often interact with and participate in enterprise-organized online service innovation activities (see Table 1 for details). The items’ and constructs’ reliability and validity were re-tested in accordance with the confirmatory study (see Tables 2, 3).

**Table 1:** Descriptive statistical analysis.

		Frequency	Percentage	Effective percentage	Cumulative percentage
Education level	Below high school	17	5.54	5.54	5.54
	junior college	5	1.63	1.63	7.17
	undergraduate	282	91.86	91.86	99.02
	Graduate and above	3	0.98	0.98	100
	Total	307	100	100	
Participation frequency	Seldom	97	31.60	31.60	31.60
	Sometimes	65	21.17	21.17	52.77
	Regular	120	39.09	39.09	91.86
	Often	25	8.14	8.14	100
	Total	307	100	100	
Income level	无	283	92.18	92.18	92.18
	2000以下	11	3.6	3.6	95.77
	2000–3,000	2	0.65	0.65	96.42
	3,000–5,000	6	1.95	1.95	98.37
	5,000以上	5	1.63	1.63	100
	Total	307	100	100	
Age	20 years and under	9	2.93	2.93	2.93
	21–30	280	91.21	91.21	94.14
	31–50	6	1.95	1.95	96.09
	50–60	12	3.91	3.91	100
	Total	307	100	100	

*n* = 392.

**Table 2:** Total variance of interpretationIngredients.

	Initial eigenvalue			Extract sum of squares loading			Rotation sum of squares loading		
	Total	% of variance	Cumulative%	Total	% of variance	Cumulative%	Total	% of variance	Cumulative%
1	6.086	27.66	27.662	6.086	27.662	27.662	2.846	12.936	12.936
2	2.835	12.88	40.55	2.835	12.888	40.55	2.584	11.746	24.682
3	2.007	9.12	49.673	2.007	9.123	49.673	2.383	10.832	35.514
4	1.822	8.28	57.955	1.822	8.283	57.955	2.294	10.429	45.943
5	1.435	6.521	64.476	1.435	6.521	64.476	2.123	9.648	55.591
6	1.106	5.028	69.504	1.106	5.028	69.504	2.079	9.452	65.043
7	1.068	4.855	74.359	1.068	4.855	74.359	2.05	9.316	74.359
8	0.681	3.096	77.455						
9	0.581	2.643	80.098						
10	0.5	2.275	82.373						
11	0.487	2.212	84.585						
12	0.45	2.044	86.63						
13	0.442	2.009	88.638						
14	0.396	1.799	90.437						
15	0.368	1.673	92.11						
16	0.334	1.517	93.626						
17	0.303	1.378	95.004						
18	0.253	1.149	96.153						
19	0.236	1.072	97.225						
20	0.218	0.99	98.215						
21	0.202	0.92	99.134						
22	0.19	0.866	100						

**Table 3:** Measurement items, factor loading, and Cronbach’s α coefficient for each variable.

Latent variable	Survey questions	Factor load	Cronbach’s α
KS	KS1:Enterprises frequently provide and share resources online to help me in developing new ideas or implementing new applications	0.779	0.808
	KS2:I often provide enterprises with information about myself and what I know. Additionally, other customers often share information with one another rather than enjoying it alone.	0.672	
	KS3:I frequently exchange information with other customers.	0.742	
VP	VP1: I have a certain right to comment on the interactive network	0.756	0.877
	VP2: I have a specific choice in the interactive network.	0.883	
	VP3: I have certain decision-making power in the interactive network.	0.746	
TS	TS1:My work consists of a single task.	0.665	0.795
	TS2:There are clearly define working standards for the work in which I am involved.	0.870	
	TS3: The project in which I participated has clear objectives.	0.871	
	TS4:My work is always geared toward developing new services and proposing novel solutions.	0.701	
CE	SE1: I think I am a person with inventiveness.	0.833	0.895
	SE2:I believe that my thoughts and actions are creative and original.	0.801	
	SE3:I believe I am knowledgeable about service innovation projects and have my own unique opinions.	0.686	
	SE4:I think I possess extensive professional knowledge regarding enterprise online innovation (about technology, resources, market understanding, product design, etc.).	0.619	
PM	PM1:I am excited to participate in enterprise service innovation online.	0.913	0.806
	PM2: Participating online in enterprise service innovation fills me with joy.	0.918	
	PM3: I am excited to be a part of online enterprise service innovation.	0.881	
	PM4: Participating in enterprise service innovation online is appealing to me.	0.81	
CC	CC1: For enterprises, the customer’s idea has a certain practicability and operability.	0.697	0.895
	CC2: The customer’s service process design scheme has a certain level of practicability and operability for enterprises.	0.634	
	CC3:For enterprises, the customer’s service product design scheme proposed has a certain practicability and operability.	0.846	
CKM	CKM1: When the co-creation project matches the customer’s knowledge, the idea put forward by the customer has certain practicability and operability	0.711	0.763
	CKM2: When the co-creation project matches the customer’s knowledge, the service process design scheme proposed by the customer has certain practicability and operability.	0.876	
	CKM3: When the co-creation project matches the customer’s knowledge, the service product design scheme proposed by the customer has certain practicability and operability	0.866	

### Variable measurement

The variables in this study are primarily measured using the domestic and foreign mature scale, with appropriate adjustments made for the Chinese context in light of the study’s actual problems (see Table 3 for details). The questionnaire utilized the standard five-point Likert scale. Respondents completed a structured questionnaire in which they were asked to rate their level of agreement with various statements. To quantify the factors affecting innovation perception climate, we employ the key scales developed by West (1987) and Foss et al. (2011).

The specific measurement of each variable is as follows. The innovation atmosphere is divided into three dimensions: knowledge sharing, virtual empowerment, and task orientation. There were 10 survey questions, which corresponded to the following categories: KS1-KS3 and VP1-VP3. We adopted Phillips’ (1997) to measure creative self-efficacy, which correspond to four questions: CE1-CE4 (see Table 3). We adopted Hermes and Riedl (2020) to measure positive emotional experience with four questions, corresponding to PM1-PM4. Furthermore, this study used Woodman et al. (1993) customer creativity scale with three questions (CC1-CC3) in Table 3 We included four control variables because the prior empirical work has suggested that “education level,” “participation frequency,” “income level,” and “age structure” have potential influence on creativity.

### Common method bias

Because cross-sectional data (such as the one collected in this study) is more likely susceptible to common method bias and variation. When designing the questionnaire, we took some precautions against this by placing the dependent variables following the independent variables in order to reduce, if not completely eliminate, the effects of consistency artefacts. In addition, we used the Harman single-factor method, which is based on confirmatory factor analyses, to determine the extent to which common method bias could affect the outcomes (Podsakoff et al., 2003). To address the issue of common method variance, we examine the fit of a single-factor model in which all items are loaded onto a single factor.

The factor analysis results reveal a total of seven dimensions with an eigenvalue larger than 1 and a total explanatory power of 74.395%. The variance of the seven variables ranged between 9.36 and 12.936%. Each dimension has an average explanatory power of 10.62%, and a standard deviation of 1.62% (see Table 2). The maximum explanatory power of a single factor is less than two standard deviations (24%), and the lowest factor explanation ability was less than 2 standard deviations. Hence, we conclude that the explanatory variation of the seven factors is average. The majority of the explanatory variation was confined to a single factor and our empirical study is not affected by the common method bias.

## Data analysis and results

### Reliability and validity analysis

We used SPSS22.0 and AMOS17.0 to perform confirmatory factor analysis to examine the reliability and validity of the measurement model. The result shows that Cronbach’s coefficient ranged between 0.76 and 0.89, all values were greater than 0.7, indicating the scales have high internal consistency, and reliability of the scale has passed the test (Table 3). Furthermore, the individual item factor loadings ranged from 0.61 to 0.91 (higher than the minimum standard value of 0.5) and were all significant, indicating preliminary evidence for the measurement model’s convergent validity^7^.

In addition to this, the composite reliability (CR) ranged between 0.69 and 0.88, exceeding 0.6 threshold value, suggesting that the items had good internal consistency (see Table 4). Furthermore, the average variance extracted (AVE) for each construct was greater than the threshold level of 0.5 (with only exception of KS) indicating that each variable had good convergence validity. In summary, the reliability and validity of our measurement satisfied the requirements.

**Table 4:** Correlations and AVE.

			Construct							
Construct	Mean	STD	CR	VP	KS	TO	CKM	CE	PE	CC
VP	5.656	0.939	**0.77**	**0.721**						
KS	5.372	0.996	0.69	0.51**	**0.648**					
TO	5.551	0.886	0.75	0.43**	0.57**	**0.714**				
CKM	5.253	0.971	0.78	0.28**	0.30**	0.33**	**0.735**			
CE	5.427	0.898	0.86	0.29**	0.22*	0.21**	0.29**	**0.781**		
PE	5.476	0.884	0.88	0.21**	0.27**	0.25**	0.24**	0.33**	**0.842**	
CC	5.532	0.819	0.86	0.21**	0.32**	0.25**	0.15**	0.27**	0.34**	**0.787**

**p < 0.05; *p < 0.1 (two-tailed test), and the value on the diagonal is the square root of AVE.

To determine the discriminant validity of the constructs, we used Fornell and Larcker's (1981) method, which states that if the standardized factor loading is at least 0.50, the average is 0.50 * 0.50, which equals 0.25—a value considered acceptable by Hair et al. (2006). The square root of the AVE of all variables are greater than the estimated intercorrelations among all constructs, and this provides support for discriminant validity (Table 4).

We further assessed the model’s fitness using construct validity, which is primarily based on absolute fit index and incremental fit index as χ^2^/df = 2.56 < 3, as well as RMSEA = 0.063 < 0.08, SRMR = 0.0622 < 0.08, IFI = 0.902, CFI = 0.901, and TLI = 0.932, all of which exceeded 0.9, demonstrating the measurement model’s better goodness-of-fit.

**Figure 2:**
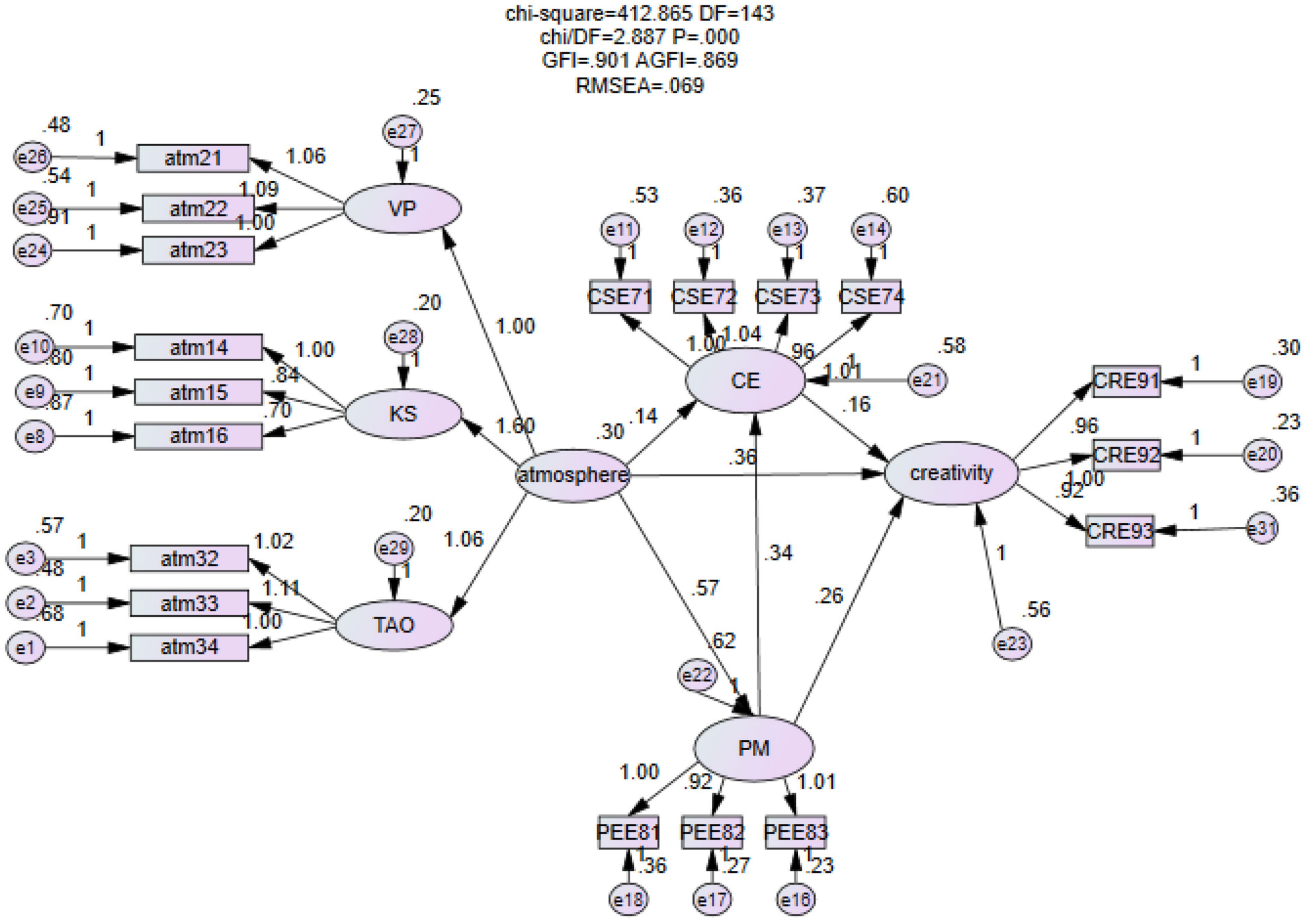
Structural equation model analysis.

In order to structure the sample data for Equation model analysis, we employ the amos22.0 software. Positive emotion self-efficacy is added to the initial model because the path coefficient is 0.34 and the *p* value is less than 0.05, indicating that positive emotion has a positive effect on innovation self-efficacy. The corrected model and results of the analysis are displayed in the Figure 2. The normalised path coefficient between all explicit variables and latent variables exceeds 0.5, as does the C R. All of the values are greater than 1.96, the model fits well, and the pertinent hypotheses have been verified.

### Main results

**Figure 3:**
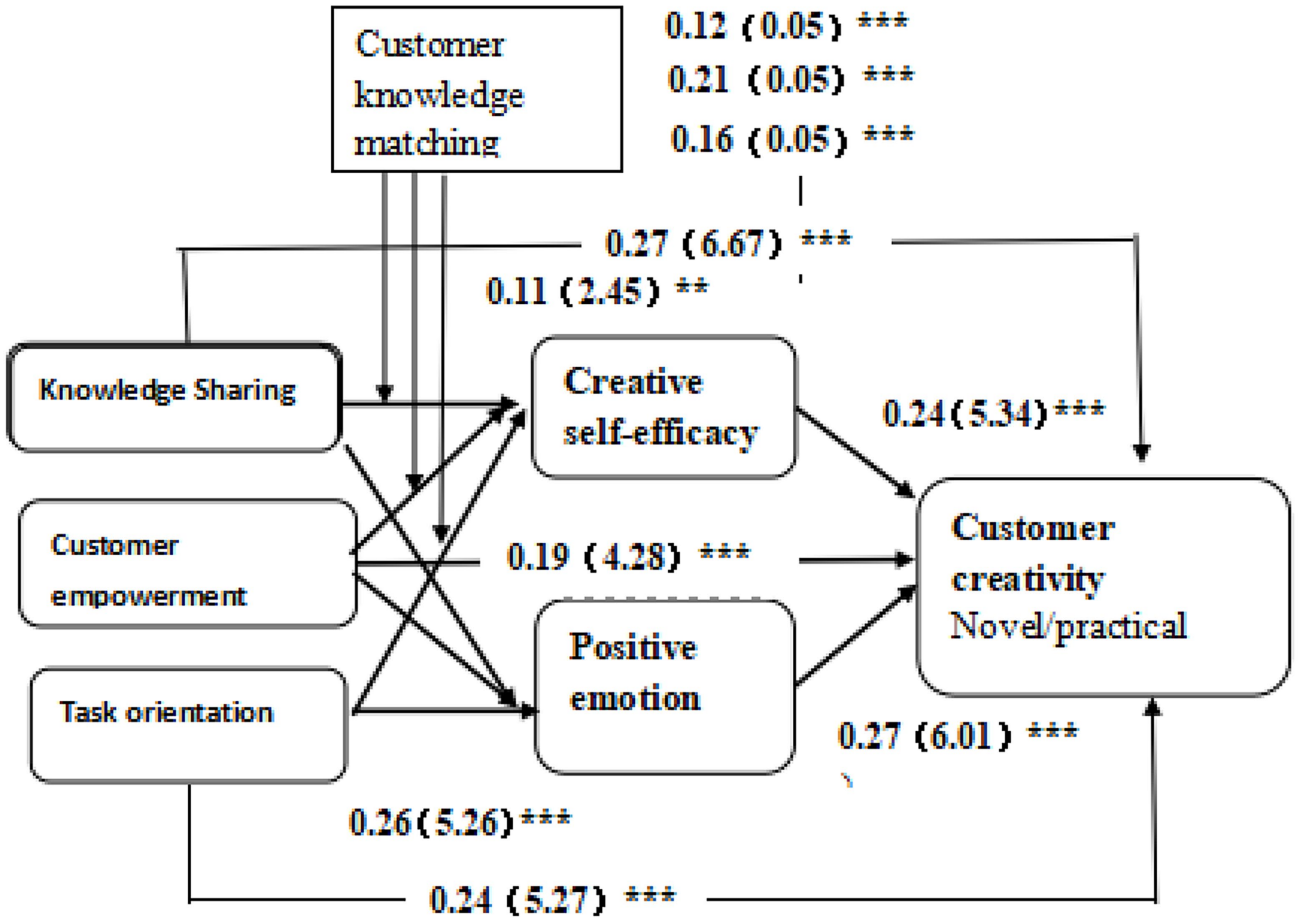
Research model verification.

We used the bootstrap method (Preacher and Hayes, 2004; Hayes, 2017) to analyze Direct effect (Column A) and total effect (Column B) of innovation environment and customer creativity (test of Hypotheses H1a-H1c), as shown in Table 5 and Figure 3. We found that knowledge sharing in an enterprise’s online community has a significant positive impact on customer creativity (β = 0.26, *p* < 0.001). Thus, hypothesis H1a was accepted. Moreover, the reported results indicate that virtual empowerment in an enterprise’s online community has a significant positive impact on customer creativity (β = 0.20, *p* < 0.001), indicating that Hypothesis H1b was supported. Finally, we found that task orientation in an enterprise’s online community has a significant positive impact on customer creativity (β = 0.24, *p* < 0.001). Therefore, Hypothesis H1c was supported.

**Table 5:** The mediating effect of creative self-efficacy and positive emotion.

The mediating effect of creative self-efficacy						The Mediating effect of positive emotion					
Hypothesis	Variable	Direct effect and total effect	Indirect effect(Column B)			Hypothesis	Variable	Direct effect and total effect	Indirect effect(Column B)		
		Β(SE)	Sobel test	Bootstrap	MacKinnon PRODCLIN2			Β(SE)	Sobel test	Bootstrap	MacKinnon PRODCLIN2
H2a	CRT on VP	0.20*** (0.04)	Value = 0.03	M = 0.03	M = 0.031	H3a	CRT on VP	0.19***(0.04)	Value = 0.06	M = 0.058	M = 0.058
	CE on VP	0.13*** (0.05)	SE = 0.01	SE = 0.013	SE = 0.013		PM on VP	0.20*** (0.05)	SE = 0.02	SE = 0.019	SE = 0.018
	CRT on CE, controlling for VP	0.24*** (0.04)	*z* = 2.368	LL95%CI = -0.008	LL95%CI = 0.007		CRT on PM, controlling for VP	0.29*** (0.05)	*z* = 3.54	LL95%CI = -0.025	LL95%CI = 0.027
	CRT on VP, controlling for CE	0.17*** (0.04)	*p* = 0.02	UL95%CI = 0.060	UL95%CI = 0.059		CRT on VP, controlling for PM	0.13*** (0.04)	*p* = 0.00	UL95%CI = 0.098	UL95%CI = 0.096
H2b	CRT on KS	0.26*** (0.04)	Value = 0.02	M = 0.024	M = 0.024	H3b	CRT on KS	0.27*** (0.04)	Value = 0.06	M = 0.062	M = 0.06
	CE on KS	0.11***(0.05)	SE = 0.01	SE = 0.012	SE = 0.012		PM on KS	0.24*** (0.04)	SE = 0.02	SE = 0.019	SE = 0.016
	CRT on CE, controlling for KS	0.22*** (0.04)	*z* = 2.17	LL95%CI = 0.005	LL95%CI = 0.003		CRT on PM, controlling for KS	0.25*** (0.05)	*z* = 3.95	LL95%CI = 0.029	LL95%CI = 0.032
	CRT on KS, controlling for CE	0.24*** (0.04)	*p* = 0.03	UL95%CI = 0.051	UL95%CI = 0.050		CRT on KS, controlling for PM	0.20*** (0.04)	*p* = 0.00	UL95%CI = 0.102	UL95%CI = 0.094
H2c	CRT on TO	0.24***(0.05)	Value = 0.05	M = 0.047	M = 0.048	H3c	CRT on TO	0.24*** (0.05)	Value = 0.07	M = 0.07	M = 0.07
	CE on TO	0.23***(0.05)	SE = 0.02	SE = 0.015	SE = 0.016		PM on TO	0.26*** (0.05)	SE = 0.02	SE = 0.021	SE = 0.019
	CRT on CE, controlling for TO	0.21***(0.05)	*z* = 3.197	LL95%CI = 0.023	LL95%CI = 0.021		CRT on PM, controlling for TO	0.27*** (0.05)	*z* = 3.927	LL95%CI = 0.036	LL95%CI = 0.037
	CRT on TO, controlling for CE	0.19*** (0.05)	*p* = 0.00	UL95%CI = 0.081	UL95%CI = 0.083		CRT on TO, controlling for PM	0.17*** (0.05)	*p* = 0.00	UL95%CI = 0.118	UL95%CI = 0.111

Standard errors in parentheses. Unstandardized regression coefficients and bias-corrected CI are reported. Bootstrap sample size = 5,000. LL is the lower limit; UL is the upper limit; CI is the confidence interval. CRT = customer creativity, PM = Positive emotions, VP=Virtual Empowerment, KS=Knowledge sharing, TO = Task orientation, CE = Creative self-efficacy. Unstandardized regression coefficients, and bias-corrected CI are reported. Bootstrap sample size = 5,000. LL is the lower limit; UL is the upper limit; CI is the confidence interval.

*** *p* < 0.01;

* *p* < 0.1.

To analyze the mediating effects of both creative self-efficacy (Hypothesis H2a-H2c) and positive emotion (Hypothesis H3a-H3c), we used bootstrap method at a 95% confidence interval with 5,000 bootstrap samples (see Table 5). The reported results reveal that the mediating test did not contain a value of 0 within the 95 percent confidence interval. With respect to hypothesis H2a and H3a, we found that both creative self-efficacy (llci = 0.005, ULCI = 0.051) and positive emotion (llci = 0.029, ULCI = 0.102) mediate the effect of online innovation environment of knowledge sharing on customer creativity. Hence, hypothesis H2a and H3a were accepted. Similarly, the reported findings indicate that creative self-efficacy (llci = 0.008, ULCI = 0.060) and positive emotions llci = 0.025, ULCI = 0.098) mediate the relationship between customer creativity and the online innovation climate of virtual empowerment, lending support to Hypothesis H2b and H3b, respectively. Finally, our findings show that the relationship between task-orientation online innovation climate and customer creativity is mediated by both creative self-efficacy (llci = 0.023, ULCI = 0.081) and positive emotion (llci = 0.036, ULCI = 0.118). Hence, both Hypothesis H2c and H3c were also supported.

### The moderating effect of customer knowledge matching

This study further investigated the moderating effects of customer knowledge matching on the association between three types of innovative atmosphere (knowledge sharing, Virtual empowerment, and task orientation) and both customer creativity and positive emotion (see Table 6). The reported in results Table 6 suggest that customer knowledge matching moderates the effect of three types of innovative atmosphere and customer creativity (*p* < 0.01). However, the relationship between an innovative environment and positive emotions is not moderated by customer knowledge (*p* > 0.01). In other words, despite their high customer knowledge matching, high knowledge sharing, virtual empowerment, and task orientation, they will not generate more positive emotions (become happier), which will negatively impact customer creativity.

**Table 6:** Results for moderated mediation.

	Dependent Variable: CE			Dependent Variable: PM		
	Model 1a	Model 2a	Model 3a	Model 1a	Model 2a	Model 3a
	KS on CRT through CE	VP on CRT through CE	TO on CRT through CE	KS on CRT through PM	VP on CRT through PM	TO on CRT through PM
KS	0.03*** (0.05)			0.19*** (0.05)		
GKM	0.25*** (0.05)			0.18*** (0.05)		
KS × CKM	0.12*** (0.05)			−0.06 (0.04)		
VP		0.06*** (0.045)			0.15*** (0.05)	
CKM		0.25*** (0.05)			0.20** (0.05)	
VP × CKM		0.21*** (0.05)			−0.06 (0.005)	
TO			0.18*** (0.05)			0.20*** (0.05)
CKM			0.23*** (0.05)			0.17*** (0.05)
TO × CKM			0.16*** (0.05)			0.02 (0.05)

*n* = 392. Control variables include age class, educational level, income level, and frequency. Unstandardized regression coefficients are reported. The level of confidence for all confidence intervals is 95%. Bootstrap sample size = 5,000.

**Figure 4:**
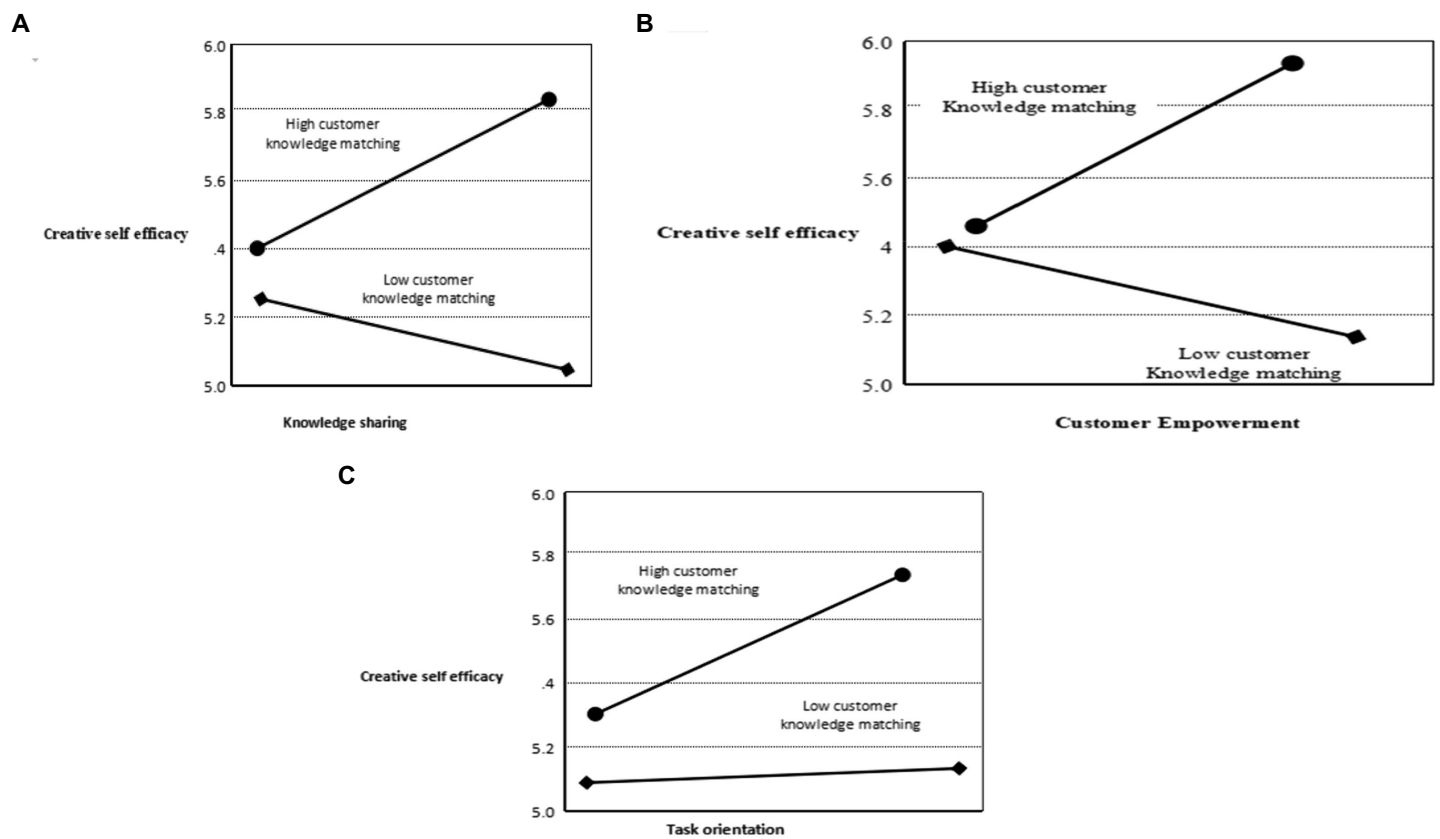
The mediating effect of customer knowledge matching. **(A)** The moderating effect of customer knowledge matching on the relationship between knowledge sharing and creative self-efficacy. **(B)** The moderating effect of customer knowledge matching on the relationship between virtual empowerment and customer creativity. **(C)** The moderating effect of customer knowledge matching on the relationship between task orientation and customer creativity.

To gain additional insight, we test the conditional indirect effect by performing PROCESS (Hayes, 2017) to determine the extent to which customers knowledge matching moderates the mediating effect of creative self-efficacy on knowledge sharing innovation atmosphere and customer creativity, as well as on virtual empowerment, innovation climate and customer creativity, and on task orientation, innovation atmosphere, and customer creativity (Hypothesis H4a- Hypothesis H4c; see Figure 4; Appendix I).

The reported results suggest that the indirect effect of knowledge sharing on customer creativity is high (0.03) when it interacts with a high level of customer knowledge matching [+1 SD, p < 0 0.05, 95% bootstrapped CI = (0.004, 0.070)], and weaker (−0.02) when it interacts with low customer knowledge matching [−1 SD, p < 0.001; 95% bootstrapped CI = (−0.054, −0.005)]. Hence, Hypothesis 4a is supported (Figure 4A). This implies that customers’ knowledge matching moderates the mediating effect of creative self-efficacy on knowledge sharing and innovation, as well as customer creativity. In addition to this, we find that the indirect effect of virtual empowerment on customer creativity is 0.06 when customer knowledge matching is high (+1 SD, *p* < 0.05, 95 percent bootstrapped CI = [0.026, 0.112]); however, the indirect effect becomes −0.03 when customer knowledge matching is low (−1 SD, p 0.001; 95 percent bootstrapped CI = [−0.069, −0.007]). Hence, Hypothesis 4b holds up (Figure 4B), indicating that customers’ knowledge matching moderates the mediating effect of creative self-efficacy on virtual empowerment, innovation climate, and customer creativity. Finally, the indirect effect of task orientation on customer creativity is high (0.069) when it interacts with high customer knowledge matching (+1 SD, p.05, 95 percent bootstrapped CI = [0.034,0.120]), and weaker (0.004) when it interacts with low customer knowledge matching (−1 SD, p < 0.001; 95 percent bootstrapped CI = [0.009,0.106]). Therefore, Hypothesis 4c is supported implying that customers’ knowledge matching acts as a moderator for the effect of creative self-efficacy on task orientation, innovation climate, and customer creativity.

### Robustness check

In order to investigate and test the reliability of regression results, this study uses independent variable partial substitution method and test method substitution method. Combined with the existing studies (Albar et al., 2012), we selected online incentive (IS) as the proxy variable of Customer Empowerment to retest the mediation effect in the model and found that results were consistent. In addition, three common methods Sobel test / bootstrap / MacKinnon are used to test the mediating effect, and the significant results are also consistent (see Table 7). Therefore, the research conclusion of this study were relatively robust.

**Table 7:** The mediating effect of creative self-efficacy.

	Direct effect and total effect				Indirect effect		
	β	SE	*t*	*p*	Sobel test	Bootstrap	MacKinnon PRODCLIN2
CRT on IS	0.38	0.04	8.87	0.00	Value = 0.04	M = 0.037	M = 0.036
CE on IS	0.20	0.05	4.09	0.00	SE = 0.01	SE = 0.014	SE = 0.012
CRT on CE, controlling for IS	0.18	0.04	4.32	0.00	*z* = 2.93	LL95%CI = 0.015	LL95%CI = 0.015
CRT on IS, controlling for CE	0.34	0.04	8.00	0.00	*p* = 0.00	UL95%CI = 0.069	UL95%CI = 0.063

## Discussions and implications

### Discussion and conclusion

This study aimed at investigating how innovative environment of online customer participation on customer participation in service innovation affects customer creativity, with a particular emphasis on the effect of an online innovation environment that addresses three basic psychological needs of customers: customer empowerment, task orientation and knowledge sharing. Additionally, this study attempted to quantify how creative self-efficacy and, positive emotions mediate the effect of, enterprise innovation environment customer creativity, and whether customer knowledge matching moderates the mediating effect of creative self-efficacy and positive emotions on the relationship between customer creativity and innovation environment. In doing so, it constructs the research model using social cognitive theory and flow theory and proposes relevant research hypotheses. The research model and hypothesis are validated through data analysis and the research results are discussed.

This study contributes by attempting to investigate the formation mechanism by which customer creativity is formed from a psychological and social cognition perspective, and by partially elucidating the driving mechanism of customer creativity in a consumption context.

The contribution of this study is to try to explore the formation mechanism by which customer creativity is formed from the perspective of psychological and social cognition perspective, and to partially explain the driving mechanism of customer creativity in a consumption situation. As, customer creativity varies according to customer and innovation task participation situation. Therefore, not all customer participation in innovation behavior has a positive impact on enterprise innovation performance. Online enterprises need to create an innovation culture that values knowledge sharing, independent empowerment, task orientation and timely incentive, as well as manage customers in a classified and hierarchical manner through long-term observation and tracking. This study provides insight and recommendations for managing customer participation mobile Internet environment. Specifically, the main conclusions of the study are as follows:

First, the innovative atmosphere of customer online participation in service innovation encourages customers to exert and promote their customer creativity (H1a, H1B, and H1C). These findings suggest that an innovation environment that encourages knowledge sharing in an enterprise’s online community can encourage customer to generate new ideas in order to improve their personal creativity. In addition to this, customer participation in virtual community with free knowledge flow, clear task orientation and sufficient independent authorization not only improves customer creativity but also helps enterprises in obtaining relevant knowledge such as customer needs and reduce barriers to knowledge sharing (Wang, 2022).

Second, creative self-efficacy acts as a partial mediator between innovation environment and user creativity (H2A, H2B, H2C). According to Bandura’s (2001) social cognitive theory, self-efficacy can either strengthen or weaken an individual’s motivation level. The study discovered that individuals with a high level of innovative self-efficacy will choose more challenging tasks. Once they start to act, they will exert more efforts and persist for a longer period of time. When they encounter setbacks, they can quickly recover. Therefore, it is capable of developing innovative products or services.

Third, positive emotions mediate the effect of online innovation atmosphere on customer creativity. Customer participation is driven by a sense of pleasure and self-improvement in the process of participation (H3a, H3b, and H3c). In other words, the experience of internal self-improvement and satisfaction is the primary reason for increasing customer stickiness and deepening and intensifying customer participation. The central principle of creativity composition theory is that people are most creative when they are motivated by their own interests, enjoyment, satisfaction and challenges of their work rather than by external incentives (Amabile, 1985). Because creativity necessitates a higher level of intrinsic motivation, customers should be encouraged to participate in virtual community in order to achieve higher levels of creative work outcomes. Intrinsic motivation is an important factor influencing customer participation because such positive beliefs motivate individuals to collaborate and share knowledge with one another for maximum benefit. Liu and Zhang (2007), for example, argued that individual internal motivation is critical to innovation performance and has a positive impact on both idea generation and execution. Customers with high internal motivation can also enjoy the process of completing their tasks (Nguyen et al., 2019).

Fourth, while the degree of customer knowledge matching moderates the mediating effect of creative self-efficacy on innovation atmosphere and customer creativity, it does not moderate the mediating effect of positive emotion on innovation atmosphere and customer creativity (H4a, H4b, H4c). From a logical standpoint, when customers participate in the enterprise, they primarily participate through knowledge transfer, assisting the enterprise in improving service innovation performance. These findings imply that customer participation will improve the transfer of customer knowledge during the process of enterprise service innovation, thereby facilitating the interaction between customers and enterprises. Thus, service innovation activities are required to be carried out by businesses by acquiring and applying customer knowledge, thereby improving their service innovation performance (Wang et al., 2020). The reason could be that increased user empowerment is positively related to positive emotions, that is, increased empowerment results in increased participation emotions (happiness, happiness, excitement, etc.), because it only involves the stimulation of emotional level, which has little to do with the degree of knowledge matching, and thus cannot affect customer creativity indirectly.

### Theoretical contributions

This study has the following main contributions. First, this study contributes to the literatures on customer innovation and open innovation by highlighting the role of organizational innovation in customer creativity. Previous research on individual creativity in psychology has mainly concentrated on employee creativity and leadership creativity, while research on customer creativity in the marketing segment is still in its early stages (Tierney and Farmer, 2002). The few studies on customer innovation and creativity have mainly examined the factors influencing customer creativity and innovation performance from one side, such as trust and psychological empowerment, but do not grasp them as a whole. What is more, previous research has used social cognitive theory to investigate the effects of creative role identity and creative self-efficacy on employee creativity (Tierney and Farmer, 2002), and other studies have measured the role of creative self-efficacy as a mediator between transformational leadership and employee creativity (Wang et al., 2014). To our knowledge, this is the first time a theoretical framework based on social cognitive theory has been used to develop a conceptual model that examines a variety of factors affecting human functioning and, consequently, the innovation climate of knowledge sharing and customer creativity.

Second, by posing the question of how customer knowledge can be used for innovation, this adds to the marketing literature. This is, as far as we know, the first study to use flow theory to lay the theoretical groundwork for understanding customer participation and its intended benefits. This study adds to the body of empirical research by explaining the mediating and moderating variables associated with the impact of the innovation climate on customer creativity outcomes. This study could help us better understand customer enterprise value co-creation behavior and modern enterprise service innovation in the post-epidemic era.

### Recommendations for managers

#### Create a free, open, and shared online interactive atmosphere for enterprises

Given the beneficial effect of an online innovation environment on customer innovation behavior and creativity, enterprises should foster a relaxed and pleasant online innovation atmosphere. For example, enterprises can implement a customer authorization strategy, allowing users to fully enjoy the innovation atmosphere of self-achievement and self-management created by authorization and reward, thereby increasing customers’ sense of self-efficacy and control, competence, and influence over the value co-creation process. This will enable the innovation atmosphere to reach and attract a broader range of consumer groups to participate in the innovation and co-construction of enterprises.

#### Invite customers to participate in service innovation activities

It is critical to invite customers to participate in innovation activities with a variety of task requirements, as this can enhance both customer creativity and enterprise innovation performance. Customers who are motivated by social sharing can be invited to participate in product trials and post-sales evaluation activities. Businesses should encourage customers to collaborate in virtual communities, improve customer communication, and coordinate and collaborate to complete tasks. It is suggested that information sharing about customer participation in virtual communities be encouraged, and that customers be given guidance on how to keep their expertise and knowledge up to date through official recommendations and other means.

#### Enhance the interactive features of online communities

The study’s finding pointed out that task orientation is extremely appealing to customers and can significantly increase their involvement in service innovation. Enterprises should effectively utilize customers’ prior knowledge when creating an innovative environment with customer participation. Additionally, they should stimulate their interest and motivation by providing timely product information and engaging interactive projects with varying levels and tasks. Businesses should enhance the adaptability, integration, and appeal of interactive channels by providing customers with more access points to virtual communities. In addition, the interpersonal interaction process would be optimized to enhance the customer’s experience of using the virtual community and to provide customers with an immersive experience. Furthermore, the interpersonal interaction process should be optimized in order to improve the customer’s virtual community experience and create an immersive experience for them. Businesses, for example, should design development participation nodes and novel interactive methods to improve (most) silent customers’ participation, the virtual community’s interactive memory system, and service innovation performance.

The implications for businesses and/or policymakers are that social capital formation within an organization is critical for knowledge sharing, implying that a social trust and interaction-based organizational culture should be promoted. By transforming their organizational culture of social trust and knowledge sharing, businesses would be able to improve their new product development and financial performance.

#### Research limitations and future directions

This research has some limitations. First, although we consider mediating variables at the cognitive (creative self-efficacy) and psychological levels (positive emotional experience). It may be inadequate and incomplete to only put the knowledge and skill variables of customer knowledge matching as the source of creativity into the moderating variables for research, which needs further testing and improvement. Second, the scales used in this study were developed in mature in foreign countries, while there is no online innovation climate scale in China. The psychological capital of different regions and countries are very different, so the definition of innovation atmosphere should also be very different. In future research, it is recommended to choose the cross-cultural context of the virtual brand community of customer innovation behavior for comparative study. It is further recommended that future studies could expand the scope of the sample to investigate the impact of cultural atmosphere factors with Chinese characteristics (such as face effect, etc.) on customer service innovation behavior. Finally, the applicability of the theory must be further verified.

## Data availability statement

The dataset used in this article will be made available upon reasonable request, further inquiries can be directed to the corresponding author/s.

## Author contributions

All authors listed have made a substantial, direct, and intellectual contribution to the work and approved it for publication.

## Funding

This research was financed by the Social Science Fund of Shandong Province (21CKFJ16).

## Conflict of interest

The authors declare that the research was conducted in the absence of any commercial or financial relationships that could be construed as a potential conflict of interest.

## Publisher’s note

All claims expressed in this article are solely those of the authors and do not necessarily represent those of their affiliated organizations, or those of the publisher, the editors and the reviewers. Any product that may be evaluated in this article, or claim that may be made by its manufacturer, is not guaranteed or endorsed by the publisher.

## Footnotes

^1^The majority of creativity research focuses on employee creativity in terms of internal organizational structure, such as the effects of transformational leadership on employee creativity. Tierney and Farmer (2002), for example, demonstrated that creative role identity and creative self-efficacy are positively related to employee creativity using a social cognitive theory perspective (Bandura et al., 1999). Creative role identity is the individual’s perception of himself as a creative person, whereas creative self-efficacy reflects the extent to which an individual believes he or she is capable of producing creative outcomes (Tierney and Farmer, 2002). Prior research has indicated that leadership style may be one of the contextual factors that boost creativity-related activities. For instance, Gong et al. (2009) for insurance agents, Walumbwa and Hartnell (2011) for a large automobile dealership, and Hon (2011) for hotel industry data all discovered that employee learning orientation and transformational leadership have a positive effect on employee creative self-efficacy.

^2^While previous studies have utilized social cognitive theory to study the effects of creative role identity and creative self-efficacy on employee creativity (Tierney and Farmer, 2002), and other measured mediating role of creative self-efficacy on the relationship between transformational leadership and employee creativity (Wang et al., 2014).

^3^A virtual community is a venue for users to exchange information for a variety of reasons.

^4^Flow is a state of mind that combines cognitive, physiological, and affective components.

^5^For example, Schreier and Prügl (2008) classified consumer knowledge into two categories: use experience and product related knowledge. While others classified it into general knowledge and professional knowledge.

^6^We followed the methods proposed by Churchill (1979), Hinkin (1998), and other scholars, for questionnaire design (for detail see Appendix I).

^7^According to Fornell and Larcker (1981), the convergent validity of measurement items in each construct should meet three conditions, that is, factor loadings, composite reliability (CR) and average variance extracted (AVE) values must be greater than 0.5, 0.8, and 0.5, respectively.

## Appendix I

The mediating effect of customer knowledge matching

**Table:**

Mediator	Moderator	Conditional indirect effect	Boot SE	LL95% CI	UL 95%CI
The mediating effect of customer knowledge matching on the relationship between virtual empowerment and customer creativity					
Creative self-efficacy	High- consistency of customer knowledge	0.06	0.022	0.026	0.112
	Low- consistency of customer knowledge	−0.03	0.016	−0.069	−0.007
The mediating effect of customer knowledge matching on the relationship between knowledge sharing and customer creativity					
Creative self-efficacy	High- consistency of customer knowledge	0.03	0.017	0.004	0.070
	Low- consistency of customer knowledge	−0.02	0.015	−0.054	−0.005
The mediating effect of customer knowledge matching on the relationship between task orientation and customer creativity					
Creative self- efficacy	High- customer knowledge matching	0.069	0.022	0.034	0.120
	Low- customer knowledge matching	0.004	0.014	0.009	0.106

Bootstrap sample size = 5,000. High = 1 SD above the mean; Low = 1 SD below the mean. The bias-corrected CI is reported.
